# Spatio-Temporal Analysis of Tuberculosis in Hamadan Province, West of Iran, from 1992 to 2013

**Published:** 2019-01

**Authors:** Salman Khazaei, Shahrzad Nematollahi, Amin Doosti-Irani, Ali Zahiri, Arash Mofarrah-Zat, Erfan Ayubi, Elham Hooshmand, Ensiyeh Jenabi, Mohammad Saatchi

**Affiliations:** 1 Research Center for Health Sciences, Hamadan University of Medical Sciences, Hamadan, Iran; 2 Men’s Health and Reproductive Health Research Center, Shahid Beheshti University of Medical Sciences, Tehran, Iran; 3 Department of Epidemiology, School of Public Health, Hamadan University of Medical Sciences, Hamadan, Iran; 4 Deputy of Health, Hamadan University of Medical Sciences, Hamadan, Iran; 5 Department of Community Medicine, School of Medicine, Zahedan University of Medical Sciences, Zahedan, Iran; 6 Iranian Research Center on Aging, University of Social Welfare and Rehabilitation Sciences, Tehran, Iran; 7 Autism Spectrum Disorders Research Center, Hamadan University of Medical Sciences, Hamadan, Iran; 8 Department of Epidemiology & Biostatistics, School of Public Health, Tehran University of Medical Sciences, Tehran, Iran

**Keywords:** Tuberculosis, Spatio-temporal analysis, Incidence, Hamadan

## Abstract

**Background:**

Tuberculosis (TB) despite being preventive and treatable still imposes a huge burden of morbidity and mortality in developing and developed countries. We aimed to investigate the spatial and geographical distribution of TB in Hamadan province during 1992–2013.

**Materials and Methods:**

This cross-sectional study was performed in Hamadan province, West of Iran using the surveillance database. We examined the trend for incidence rates of all TB forms including Smear Positive Pulmonary TB (SPPT), Smear Negative Pulmonary TB (SNPT) and Extra pulmonary TB (EPT) per 100,000 populations. Poisson regression model was used to estimate the standardized rates for incidence rate of all types of TB per each county.

**Results:**

In this study 3,602 TB patients including 1,359 SPPT, 987 SNPT, and 1,256 EPT were included during 1992–2013. Trend of all types of TB decreased from 1992 to 2013. The Average Annual Percent change (AAPC) for all types of TB was significantly (P<0.05) decreased, AAPC= −6.4 (95% CIs: −10.7, −1.9). Among SPPT, SNPT, and EPT incidence rates, the maximum change was related to SNPT (−11.6; 95% CIs: −24.2, 3), while it was −1.4 (−8.7, 6.4) for SPPT and −5.8 (−11.4, 0.1) for EPT.

**Conclusion:**

Our results showed that the incidence of TB in Hamadan province during a 22-year period has decreased 6.4% on average, somehow higher than the national average. Furthermore, our study showed that the risk of extra-pulmonary occurrence in western parts of the province is higher than others parts.

## INTRODUCTION

*Mycobacterium tuberculosis* -the causative agent of Tuberculosis (TB) - is one of the oldest human pathogens, which despite being preventive and treatable, still imposes a huge burden of morbidity and mortality in developing and developed countries (1). With involvement of all ages, the incidence of TB is escalated in people in economically active ages, which leads to many direct and indirect economic consequences (2, 3). According to the 2015 World Health Organization (WHO) report, more than 87% of incident cases of TB have been reported from 30 countries, of which 61% happened in Asian ones. Simultaneously, there were more than 10.4 million new cases and 1.8 million deaths attributable to TB (4). Iran is neighboring with the three most TB prevalent countries, namely Pakistan, Afghanistan, and Iraq (4). Nevertheless, the trend of TB has been decreasing since the introduction of Directly Observed Treatment, Short-course (DOTS) strategy in the country (5). According to the same year’s report of WHO, the incidence of TB in Iran decreased from 18.4 in 2000 to 13 in 2015 (per 100,000 population) (6). The global epidemiology of TB is well-studied; however, some concerns stemmed from the fact that the global distribution of TB is asymmetric. In other words, it is influenced by a pile of geographical (e.g. access to health care) and environmental (population socio-economic status, density, poverty, and health literacy level) factors (7–9). Therefore, determination of the hotspots and high-risk areas in order to identify the most important causes of TB in different parts of the world has become a milestone in prevention strategies. This approach is quite well known as “spatial Epidemiology”. It utilizes GIS (Geographic Information System) software and other spatial statistics to describe the role of environmental and geographical factors in the disease occurrence, and to select the factors that have the most impact on allocation of preventive and curative resources (10, 11). Furthermore, describing the trend of the disease over time may provide proper information on the effectiveness of various preventive and therapeutic healthcare services, while it can also predict the future rate and frequency of the disease for policy health makers. An investigation of the trend of TB in Iran conducted by Arsang et al. showed that since 1992 the trend has decreased due to the improved sanitation and health indices. The authors also reported the lack of non-linear models to address non-linear trends of the disease in the country as the main challenge of their study (12).

Hamadan province located in the west of Iran with a population of 1,750,000 comprises of nine counties, and variety of ethnic and climate conditions. The reported pattern of TB in the province is decreasing. However, in one work by Khazaei et al. it was reported that incidence of TB in provincial level increased from 3.4 in 2005 to 7.4 in 2011 (13). Given the importance of the temporal trend of TB and application of non-linear models to determine the hotspots, the present study investigated the spatial and geographical distribution of TB in Hamadan province during 1992–2013.

## MATERIALS AND METHODS

### Study area

This cross-sectional study was performed in Hamadan province, West of Iran using the surveillance database. All provincial registered TB patients from 1992 to 2013 were included in the study.

### Data source

We examined the trend for incidence rates of all TB forms including Smear Positive Pulmonary TB (SPPT), Smear Negative Pulmonary TB (SNPT) and extra pulmonary TB (EPT) per 100,000 populations. TB cases were defined according to the WHO and national TB guideline in Iran (14). Expatriate patients such as Afghans, non-native patients, and imported cases were excluded from the study.

### Statistical methods

#### Temporal pattern

Due to non-constant trend for types of TB over this time period, segmented analysis was used. This model assumption is that changes in the rates are constant over each time segment called change points, but varies with other time segments (15). In this study the trend, Annual Percent change (APC), and Average Annual Percent change (AAPC) of all types of TB were considered as independent variable and incidence rates for types of TB (per 100,000 population) during 1992–2013 was considered as dependent ones. APC is a way to measure trends of disease over time, and AAPC is the summary of trend and determines the interval of years, to determine the summery statistics of trend. These statistical analyses had done with Joinpoint software, V3.5.1.

### Spatial pattern

Using Poisson model, standardized rates were estimated for incidence rate of all types of TB per county. In a given population *i*, observed number of events depends on (1) area-specific Relative Risk (*RR_i_*) and (2) number of events that it have a Poisson distribution with mean of *E_i_RR_i_*. Expected events calculated as follows:

$$E_{i}=n_{i}\left(\frac{\sum_{i}y_{i}}{\sum_{i}n_{i}}\right),\;i=1,2,\ldots .I$$

Where *n_i_* is population in county*i*, *y_i_* is observed number of events in county *i*. *RR_i_* is estimated as dividing the observed number of event in county by expected event in that county.

Spatial analysis carried out by SaTScan version 9.4. The level below 0.05 was considered as significant for all statistical tests.

## RESULTS

In this study 3,602 TB patients including 1,359 SPPT, 987 SNPT, and 1,256 EPT were included during 1992–2013. Trend of all types of TB decreased from 1992 to 2013 (Table 1). The highest and lowest incidence of all types of TB were related to 1992 (25.62) and 2006 (5.38), respectively. The highest and lowest incidence rate of SPPT were related to 1995 (5.49) and 2006 (2.43), respectively. For SNPT the maximum and minimum incidence rate were related to 1992 (15.28) and 2011 (0.67), respectively. In the case of EPT the maximum and minimum incidence rate were in 1992 (6.24) and 2005 (1.8), respectively (Table 1).

**Table 1.** Incidencr rate of all TB types in Hamadan province 1992–2013

**Year**	**SPPT**		**SNPT**		**EPT**		**All form**	

	**Frequency**	**Incidence**	**Frequency**	**Incidence**	**Frequency**	**Incidence**	**Frequency**	**Incidence**
**1992**	69	4.1	257	15.28	105	6.24	431	25.62
**1993**	65	3.89	53	3.17	88	5.27	206	12.34
**1994**	74	4.4	65	3.87	66	3.93	205	12.2
**1995**	92	5.49	86	5.13	82	4.89	260	15.51
**1996**	62	3.67	45	2.67	101	5.99	208	12.33
**1997**	69	4.07	33	1.95	85	5.01	187	11.02
**1998**	57	3.35	37	2.18	89	5.24	183	10.77
**1999**	79	4.63	69	4.05	74	4.34	222	13.02
**2000**	70	4.14	66	3.9	69	4.08	205	12.11
**2001**	50	2.91	28	1.63	62	3.61	140	8.15
**2002**	76	4.39	16	0.93	40	2.31	132	7.63
**2003**	60	3.45	20	1.15	48	2.6	128	7.36
**2004**	63	3.62	23	1.32	40	2.3	126	7.23
**2005**	46	2.67	19	1.1	31	1.8	96	5.57
**2006**	42	2.43	16	0.93	35	2.02	93	5.38
**2007**	54	3.15	12	0.7	36	2.1	102	5.94
**2008**	65	3.7	23	1.31	38	2.17	126	7.18
**2009**	51	2.84	14	0.78	34	1.9	99	5.52
**2010**	57	3.19	34	1.9	31	1.73	122	6.83
**2011**	57	3.16	12	0.67	31	1.72	100	5.54
**2012**	46	2.62	23	1.31	41	2.34	110	6.27
**2013**	55	3.14	27	1.54	39	2.00	121	6.91

The AAPC for all types of TB significantly (p<0.05) decreased, AAPC= −6.4 (95% CIs: −10.7, −1.9). Among SPPT, SNPT, and EPT incidence rates, the maximum change was related to SNPT (−11.6; 95% CIs: −24.2, 3), while it was −1.4(−8.7, 6.4) for SPPT and −5.8 (−11.4, 0.1) for EPT, However, these changes were not statistically significant (Table 2).

**Table 2.** Number and local of change points, APC and AAPC for TB indices new cases in Hamadan, 1992–2013

**Index**	**No. of change points (Year)**	**Years**	**APC (95%CI)**	**AAPC (95%CI)**	**P value**
**SPPT incidence rate**	2 (2002, 2005)	1992–2002	−1.1 (−4.8, 2.8)	−1.4 (−8.7, 6.4)	0.7
		2002–2005	−9.7 (−45.1, 28.4)		
		2005–2013	2.4 (−5.9, 11.4)		
**SNPT incidence rate**	2 (1994, 2006)	1992–1994	−39.3 (−84,129.9)	−11.6 (−24.2, 3)	0.1
		1994–2006	−11.1 (−18.8, −2.8)		
		2006–2013	1.2 (−24.9, 36.3)		
**EPT incidence rate**	2 (1999,2002)	1992–1999	−1.3 (−6.4, 4.1)	−5.8 (−11.4, 0.1)	0.1
		1999–2002	−20 (−46.2, 18.90)		
		2002–2013	−4.1 (−7.5, −0.5)		
**All form incidence rate**	2 (1994, 2006)	1992–1994	−18.4 (−45.8, 22.8)	−6.4 (−10.7, −1.9)	<0.05
		1994–2006	−6.9 (−9.5, −4.3)		
		2006–2013	0.2 (−8.6, 9.8)		

**APC:** Annual Percent change, **AAPC:** Average Annual Percent change

**Figure 1.**
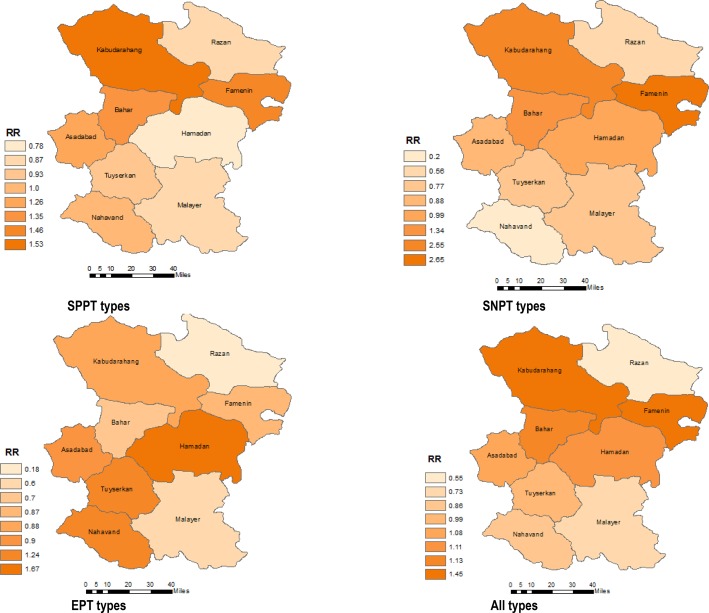
The observed by expected number (rate ratio) of TB types in Hamadan province (2005–2013)

The highest ratio for the ratio of expected number of all types of TB was related to Kabudarahang and Famenin counties. This ratio for SPPT in Kabudarahang and Famenin counties was higher compared to other counties. For SNPT higher rate ratio was related to Famenin and Kabudarahang counties, respectively. In the case of EPT, the higher rate ratio was related to Hamadan city (Figure 1).

## DISCUSSION

To our knowledge, this is the first study investigating the distribution of TB according to time and location in Hamadan province using segmented regression models and GIS software. The results showed that during 22 years (1992–2013) the annual incidence of all types of TB had decreased on average 6.4%. Consistent with this finding, MDGs achievement report issued by the WHO also shows that there was 1.5% annual decrease in the incidence of TB during 2000–2014 (16). Kazemnezhad et al. reported that while the annual trend of TB occurrence in all WHO regional offices had declined 1.4% during 1990–2010, the least decrease belonged to Eastern *Mediterranean* office (17). Using segmented regression models in Iran, Khazaei et al. showed that the 25-year trend of TB incidence (1990–2014) had decreased 1.7% each year. They also reported that the decrease in occurrence of all types of TB in Hamadan province was distinguishable compared to that in national and international levels (18). This finding indicates the satisfactory performance of the provincial surveillance system despite the detrimental effect of immigrations, population growth, and increased incidence of HIV/AIDS among drug users.

According to the TB national surveillance guideline, the rate of SPPT is the main indicator of TB status in the country. Therefore, a paucity of evidence exists regarding the descriptive and analytic epidemiology of this type of TB. Nonetheless, state of the art and up-to-dated evidence concerning time trend of the disease are still scarce.

Arsang et Al. in a study on 40-year TB trend reported that with an average annual decrease of 4.5% during 1998–2008, the incidence of SPPT in 2008 decreased tenfold compared to 1964. More in-depth investigation during the same time period (2000–2008) revealed that the highest annual decrease in provincial smear-positive TB incidence observed in Rafsanjan (AAPC=20.3%), while the lowest was belonged to Khorasan (AAPC=3.6%) (12). Our results, however, showed that the incidence of smear-positive TB during the aforementioned time did not show any significant decrease in Hamadan province and remained plateau. Due to the important contribution of SPPT cases in disease transmission, the investigation of possible causes of non-decreasing trend in Hamadan province is imperative for health system management. Increase of HIV infection, population growth especially in suburban, pilgrimage, and misdiagnosis or mistreatment of smear-positive TB cases in Hamadan province could be responsible for haltering the synchronous decreasing trend of the disease.

Investigation of the outcome of treatment in pulmonary TB cases in Hamadan province revealed that 3.4% of cases failed the treatment and recovery rate of pulmonary TB cases in Hamadan province which was lower than global projections (18).

Our results also showed that during 1994–2006 the annual incidence of smear-negative TB had decreased on average 11% (−18.8, −2.8). In addition, during 2002–2013 extra-pulmonary TB had decreased on average 4.1 (−7.5, −0.5) each year. Since the trend of smear-positive TB was plateau during the same period, the total decrease intuitively could be attributed to smear-negative and extra-pulmonary cases. Nevertheless, the total estimation of annual change in incidence was −11.6 for smear-negative and −5.8 for extra-pulmonary, yielding no significant difference (P value=0.1). Identification of high-risk areas of TB in the country empowers decision-makings of healthcare service deliveries by prioritizing financial resources, prevention and therapeutic measures and also research themes.

Our findings showed that the incidence of all types of TB in Hamadan province did not demonstrate any consistent distribution and it varied among different counties with a peak in KabodarAhang, Famenin, and Hamadan counties. This finding is consistent with a geographically heterogenic pattern for the disease reported before (5, 19).

According to our results, the risk of TB in Kabodarahang and Famenin counties was 1.45, e.g. 45% higher than expected. Specifically, the incidence of smear-positive and smear-negative TB was 53% (RR=1.53) and 166% (RR=2.66) higher than expected in Kabodarahang county. Two explanations for higher incidence of pulmonary TB in KabodarAhang and Famenin counties compared to other counties are noteworthy. Firstly, the satisfactory performance of surveillance system might lead to timely diagnosis of the cases and increase of the occurrence rates. However, synchronous performance of this system all around the country according to the national TB surveillance guideline makes this justification unlikely.

Secondly, several studies have shown that different and heterogenic distribution in national or provincial level could be due to diversity of socioeconomic status, environmental factors, or clinical conditions such as HIV infection prevalence, malnutrition, and diabetes (20, 21). Therefore, further geographical investigation of these factors seems imperative.

According to our results, the risk of TB in west of the province was higher which could be explained by neighboring this part of the province with provinces such as Kordistan and Kermanshah as the entry points from Iraq travelers. The effect of neighboring with high prevalence areas has been reported from different studies in Iran. The study of Yazdani-Charati et al. (5) in Mazandaran province showed that the incidence of pulmonary TB in cities near to high-risk province of Golestan, could change the distribution of the disease.

One of the interesting remarks of this study is the high incidence of extra pulmonary TB in Hamadan city compared to other counties. We found that extra pulmonary TB occurred 67% more than expected in Hamadan city. Due to different clinical manifestations of this type of TB, this is possible that the lack of expert physicians and inaccessible health-care services in other counties could lead to under-reporting. This finding is consistent with the findings of Tabatabaee et al. (19) in Fars province showing that the incidence of extra-pulmonary TB in Shiraz city is higher than that in other cities of the province. Moreover, accounting one third (37%) of all reported cases of TB as extra pulmonary indicates the satisfactory performance of the case finding system in the provincial level.

## CONCLUSION

Our results showed that the incidence of TB in Hamadan province during a 22-year period has decreased 6.4% on average, somehow higher than the national average. Furthermore, our study showed that the risk of extra-pulmonary occurrence in western parts of the province is higher than others, while this type of the disease in Hamadan city as a canon of political and geographical area in the country has significant implications.
